# Functional characterization of *Trip10 *in cancer cell growth and survival

**DOI:** 10.1186/1423-0127-18-12

**Published:** 2011-02-07

**Authors:** Chia-Chen Hsu, Yu-Wei Leu, Min-Jen Tseng, Kuan-Der Lee, Tzen-Yu Kuo, Jia-Yi Yen, Yen-Ling Lai, Yi-Chen Hung, Wei-Sheng Sun, Chien-Min Chen, Pei-Yi Chu, Kun-Tu Yeh, Pearlly S Yan, Yu-Sun Chang, Tim H-M Huang, Shu-Huei Hsiao

**Affiliations:** 1 Human Epigenomics Center, Department of Life Science, Institute of Molecular Biology and Institute of Biomedical Science, National Chung Cheng University, Chia-Yi, Taiwan; 2 Chang Gung Memorial Hospital, Chia-Yi, Taiwan; 3 Division of Neurosurgery, ChangHua Christian Hospital, ChangHua, Taiwan; 4 Department of Pathology, ChangHua Christian Hospital, ChangHua, Taiwan; 5 Division of Human Cancer Genetics, Department of Molecular Virology, Immunology, and Medical Genetics, and the Comprehensive Cancer Center, The Ohio State University, Columbus, OH 43210, USA; 6 Graduate Institute of Basic Medical Sciences, Chang Gung University, Tao-Yuan, Taiwan

## Abstract

**Background:**

The Cdc42-interacting protein-4, Trip10 (also known as CIP4), is a multi-domain adaptor protein involved in diverse cellular processes, which functions in a tissue-specific and cell lineage-specific manner. We previously found that *Trip10 *is highly expressed in estrogen receptor-expressing (ER^+^) breast cancer cells. Estrogen receptor depletion reduced *Trip10 *expression by progressively increasing DNA methylation. We hypothesized that *Trip10 *functions as a tumor suppressor and may be involved in the malignancy of ER-negative (ER^-^) breast cancer. To test this hypothesis and evaluate whether *Trip10 *is epigenetically regulated by DNA methylation in other cancers, we evaluated DNA methylation of *Trip10 *in liver cancer, brain tumor, ovarian cancer, and breast cancer.

**Methods:**

We applied methylation-specific polymerase chain reaction and bisulfite sequencing to determine the DNA methylation of *Trip10 *in various cancer cell lines and tumor specimens. We also overexpressed *Trip10 *to observe its effect on colony formation and *in vivo *tumorigenesis.

**Results:**

We found that *Trip10 *is hypermethylated in brain tumor and breast cancer, but hypomethylated in liver cancer. Overexpressed *Trip10 *was associated with endogenous Cdc42 and huntingtin in IMR-32 brain tumor cells and CP70 ovarian cancer cells. However, overexpression of *Trip10 *promoted colony formation in IMR-32 cells and tumorigenesis in mice inoculated with IMR-32 cells, whereas overexpressed *Trip10 *substantially suppressed colony formation in CP70 cells and tumorigenesis in mice inoculated with CP70 cells.

**Conclusions:**

*Trip10 *regulates cancer cell growth and death in a cancer type-specific manner. Differential DNA methylation of *Trip10 *can either promote cell survival or cell death in a cell type-dependent manner.

## Background

Trip10 is a scaffold protein with F-BAR, ERM, and SH3 domains. Because these domains interact with diverse signaling partners, Trip10 is involved in various cellular processes including insulin-stimulated glucose uptake, endocytosis, cytoskeleton arrangement, membrane invagination, proliferation, survival, and migration, in a tissue-specific and cell lineage-specific manner. In adipocytes, Trip10 increases glucose uptake by interacting with TC-10 to regulate insulin-stimulated glucose transporter 4 (Glut4) translocation to the plasma membrane [1,2]. However, in muscle cells, Trip10 inhibits glucose uptake by increasing Glut4 endocytosis [3,4]. In natural killer cells, Trip10 regulates actin cytoskeleton dynamics by interacting with WASP protein [5,6], and regulates cytotoxicity by facilitating localization of microtubule organizing centers to immunological synapses [7]. Trip10 is also a regulator or modulator of cell survival after DNA damage [8] and in the human brain affected by Huntington's disease [9]. Trip10 expression is decreased in hepatocyte growth factor/scatter factor (HGF/SF)-mediated cell protection against DNA damage, but is significantly increased during hyperbaric oxygen-induced neuroprotection [10]. On the other hand, overexpression of Trip10 was observed in human Huntington's disease brain striatum, and neuronal Trip10 immunoreactivity increased with neuropathological severity in the neostriatum of Huntington's disease patients [9]. In addition, rat striatal neurons transfected with Trip10 exhibited increased cell death [9], suggesting that Trip10 is toxic to striatal neurons. These data demonstrate that the function of Trip10 in cell survival and growth is cell lineage-specific. These diverse and sometime opposing roles of Trip10 may be due in part to splicing variants, but equally important, they could be the result of Trip10 interaction with distinct signaling partners in different cell types.

Trip10 also appears to be involved in tumorigenesis and cancer progression. Enforced expression of *Trip10 *increases DNA damage-induced cell death in MDA-MB-453 human melanoma cells and DU-145 human prostate cancer cells [8]. However, *Trip10 *overexpression enhances pancreatic cancer cell migration by downregulating the antitumor function of ArgBP2, suggesting that Trip10 contributes to the malignancy of pancreatic cancer [11]. In epidermoid carcinoma cells, siRNA-mediated silencing of *Trip10 *strongly increases epidermal growth factor receptor levels, sustains extracellular signal-regulated kinase activation, and promotes cell cycle progression into S phase [12], which may contribute to excessive proliferation and tumorigenesis. In Epstein-Barr virus-transformed lymphoblastoid cell lines, blocking the NF-κB pathway induces apoptosis and suppresses *Trip10 *[13], suggesting that *Trip10 *activation is crucial for the proliferation and survival of lymphoblasts.

DNA methylation is an epigenetic mechanism that regulates gene expression in response to intrinsic and environmental signals under normal physiological conditions (e.g., development) and pathologic conditions (e.g., cancer) [14-17]. A cohort of methyl CpG-binding proteins is recruited specifically to methylated CpG sites, where they repress transcription by limiting the access of transcription factors to the promoter. DNA hypermethylation silences tumor suppressor genes in many cancers, and the spreading of DNA hypermethylation correlates positively with tumor progression. We previously reported that *Trip10 *is an estrogen receptor (ERα) downstream target and subject to hormone-regulated epigenetic regulation [18]. In MCF7 cells, an estrogen receptor-positive (ER^+^) breast cancer cell line, *Trip10 *is strongly expressed. Loss of estrogen receptor signaling gradually reduces *Trip10 *expression by triggering DNA methylation. Consistently, the *Trip10 *promoter is hypermethylated in ER^- ^human breast tumors, but not in ER^+ ^breast tumors.

To evaluate whether *Trip10 *function is regulated in a lineage-dependent manner, we used methylation-specific polymerase chain reaction (MSP) and bisulfite sequencing to assess DNA methylation of *Trip10 *in human primary tumor specimens and cell lines. We then overexpressed human *Trip10 *to evaluate its effect on colony formation and *in vivo *tumorigenesis in immunodeficient mice. We found that *Trip10 *is differentially methylated in different cancers. Overexpression of *Trip10 *increases colony formation and tumorigenesis of IMR-32 cells, but decreases colony formation and tumorigenesis of CP70 cells. Taken together, our results show that *Trip10 *expression in brain tumors, breast cancer, liver cancer, and ovarian cancer is regulated by DNA methylation, but the methylation level varies among these cancer types. Trip10 functions as a tumor suppressor or an oncogene, depending on the cell type in which it is expressed.

## Methods

### Cell culture

IMR-32 neuroblastoma and U87 glioma cells were grown in Dulbecco's modified Eagle's medium, CP70 ovarian carcinoma cells were grown in RPMI 1640, MCF7 breast adenocarcinoma and HepG2 liver carcinoma cells were grown in Minimum Essential Medium (MEM), and MDA-MB-231 breast adenocarcinoma cells were grown in Leibovitz's L-15. All cell cultures were supplemented with 10% fetal bovine serum, 2 mM L-glutamine, and 100 μg/ml penicillin/streptomycin. Human bone marrow-derived mesenchymal stem cell (MSC) isolation and culture were performed as described previously [19]. Expansion medium consisted of MEM-α and 20% newborn calf serum supplemented with 100 μg/ml penicillin/streptomycin and 2 mM L-glutamine. Cells were allowed to adhere overnight at 37°C in 95% O_2_/5% CO_2_. Thereafter, the culture medium was changed twice weekly. Cells were passaged at 90% confluence. All reagents were purchased from Invitrogen.

### Cloning of the human Trip10 promoter

Primer sequences for human *Trip10 *are listed in Additional File 1: Table S1. Total RNA from MDA-MB-231 cells was purified and reverse transcribed; the resulting cDNA was used as template for PCR amplification. Purified PCR products were ligated into a cloning vector (TOPO-TA cloning kit, Invitrogen), according to the manufacturer's protocol. Inserts were confirmed by restriction digest analysis and sequencing. *Trip10 *was then subcloned into the pcDNA3.1 vector for transfection (*pcDNA-Trip10*).

### Transfectio

The *pcDNA-Trip10 *plasmid (1 μg) was transfected into IMR-32 and CP70 cells using DMRIE-C transfection reagent (Invitrogen), according to the manufacturer's instructions. Empty vectors were transfected into control cells as vehicle control. The antibiotic G418 (500 μg/ml) was added to culture medium for stable clone selection.

### Bisulfite sequencing

Genomic DNA (0.5 μg) was treated with bisulfite (Zymo), PCR-amplified, cloned, and sequenced as described by Yan et al [20]. PCR primers are listed in Additional File 1: Table S1.

### Quantitative MSP

Quantitative MSP (qMSP) was performed as described by Yan et al [20]. Universal methylated DNA (Millipore) served as positive control, and *Col2A1 *as loading control. Primers for *Col2A1 *were used to amplify serial dilutions (1/10, 1/100, and 1/1000) of control bisulfite-converted genomic DNA to generate a standard curve (Bio-Rad iQ5 real-time thermal cycler). The percentage of methylation was calculated as (florescence intensity of *Trip10 *amplification) ×100%/(florescence intensity of *Col2A1 *amplification). The 25-μl qMSP reaction contain 4 μl bisulfite-treated DNA template, 2 μl primers (each primer mix, 2.5 μM), 12.5 μl reaction buffer (2× SYBR Green real-time PCR Master Mix, Toyobo), and 6.5 μl ddH2O. The PCR primers are listed in Additional File 1: Table S1.

### Immunoblotting

Cell lysates were collected, and protein concentration was determined with a protein assay kit (Bio-Rad) using bovine serum albumin (BSA) as the standard. Proteins (40 μg/lane) was separated by gel electrophoresis and transferred to PVDF membrane. The membranes were rinsed with Tris-buffered saline Tween 20 (TBST; 20 mM Tris, 500 mM NaCl, pH7.5, 0.05% Tween 20) and blocked with 5% non-fat milk in TBST for 50 min at room temperature. After rinsing with TBST, the membrane was incubated with primary antibodies in TBST overnight at 4°C. After rinsing with TBST, the membrane was incubated with secondary antibodies for 45 min at room temperature, and then rinsed again with TBST. Membranes were incubated with chemiluminescence reagent and exposed to x-ray film.

### Immunoprecipitation

To evaluate the interactions of Trip10 with endogenous Cdc42 and huntingtin in IMR-32 cells and CP70 cells, immunoprecipitation was carried out with the Catch and Release immunoprecipitation kit (Upstate) according to the manufacturer's instructions.

### Immunostaining

Cells were fixed in 2% formaldehyde in phosphate buffered saline (PBS) and permeabilized in PBS containing 0.5% NP40. After blocking with horse serum (1:100 in PBS), the cells were incubated with primary antibodies in PBS with 3% BSA. After washing with PBS, the cells were incubated with secondary antibodies in PBS with 3% BSA. After several PBS washes, the slides were mounted with mounting medium containing 4',6-diamidino-2-phenylindole (DAPI; Vector Laboratories). The primary antibodies were anti-Cdc42 (BD Transduction Laboratories), anti-huntingtin (Chemicon), and anti-Trip10 (Abcam). Fluorescein or Texas red-conjugated anti-mouse or anti-rabbit IgG (Vector Laboratories) secondary antibodies were used for detection.

### Soft agar assay

Soft agar was made with 0.5% bottom agar and 0.3% top agar. After plating the bottom agar, cells were mixed with top agar and plated (5 × 10^4^/well). After 2 weeks of culture, cells were stained with 0.01% crystal violet, and the spheres (> 50 cells) in each well was counted.

### In vivo tumorigenesis

Mock-transfected or *Trip10-*overexpressing IMR-32 and CP70 cells (1 × 10^7 ^cells) were subcutaneously injected into 6-week-old nude mice (Narl:ICR-Foxn1nu).

### Immunohistochemistry

Tumor masses were surgically removed from nude mice inoculated with *Trip10*-overexpressing IMR-32 or CP70 cells. The tumor specimens were embedded in paraffin and cut into 4-μm sections or embedded in OCT and cut into 12-μm sections on a cryostat (Leica). Sections were stained with hematoxylin and eosin.

### Chromatin immunoprecipitation (ChIP)

ChIP assay was performed as described by Jin et al [21].

### Human subjects

Human cancer tissue collection followed IRB regulations as mandated by ChangHua Christian Hospital, Taiwan. Isolation and characterization of human MSCs were conducted according to IRB regulations at Chang-Gung Memorial Hospital, Taiwan.

### Animal studies

The use of mice followed the regulations and protocols reviewed and approved by the Institutional Animal Care and Use Committee at National Chung Cheng University.

## Results

### Trip10 is differentially methylated in human cancer cell lines and primary tumor specimens

**Figure 1:**
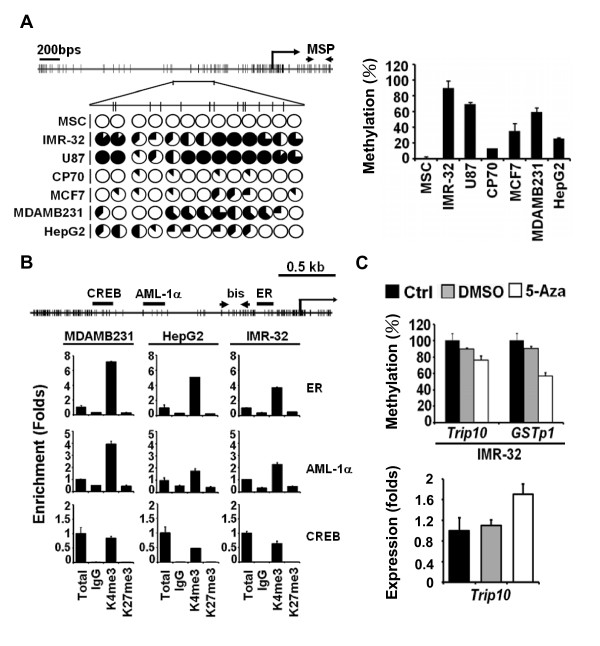
**Epigenetic regulation of *Trip10***. (A) Bisulfite sequencing (left) and qMSP (right) shows *TripP10 *methylation in various cancer cell lines. CpG locations are indicated as vertical bars in the promoter and first exon of *Trip10 *(top). Arrows mark the location of MSP primers. Open circles indicate unmethylated CpG sites, and circles filled to varying degrees reveal the percentage of methylation at specific CpG sites. Results of eight clones from each cell line are presented. For qMSP, *Col2A1 *was used as loading control. (B) H3K4me3 and H3K27me3 association at *Trip10 *promoter were demonstrated by ChIP analysis. CREB, AML-1α, and ER transcription factor binding sites are shown with individual CpG sites (short vertical bars). Arrows indicate the bisulfite sequencing region shown in (A). All three transcription factor binding sites were associated with H3K4me3, but not H3K27me3. (C) DNA demethylation. IMR-32 cells treated with 5-Aza (20 μM) or DMSO (vehicle) were analyzed by qMSP and qRT-PCR. *Col2A1 *served as loading control for qMSP, and *GAPDH *served as loading control for qRT-PCR.

We first compared DNA methylation at the *Trip10 *promoter and first exon in cancer cell lines and somatic stem cells (MSCs) from normal human adults by bisulfite sequencing and qMSP. The *Trip10 *promoter was either unmethylated or undermethylated in MSCs and CP70 ovarian cancer cells as revealed by bisulfite sequencing, but the same sequence was moderately methylated in breast cancer cells (MCF7 and MDA-MB-231) and liver cancer cells (HepG2). Heavy methylation was seen in brain tumor cells (IMR-32 and U87) (Figure 1A left, Additional File 1: Figure S1). Methylation of the *Trip10 *first exon determined by MSP was similar to the pattern observed in the promoter region, in which methylation was undetectable in MSCs, slightly methylated in CP70, moderately methylated in MCF7, MDA-MB-231 and HepG2 cells, but hypermethylated in IMR-32 and U87 cells (Figure 1A right). In our previous study, expression of *Trip10 *during MSC-to-lineage-specific differentiation is also subjected to histone medications [22], thus promoter association with histone 3 lysine 4 trimethylation (H3K4me3, active histone mark) and histone 3 lysine 27 trimethylation (H3K27me3, repressive mark) were analyzed by chromatin immunoprecipitation (ChIP). As shown in Figure 1B, all putative ER, AML-1α, and CREB binding sites on *Trip10 *promoter were enriched for H3K4me3, but not H3K27me3, confirming that *Trip10 *expression is regulated by both DNA methylation and histone modification. A comparison of endogenous *Trip10 *mRNA expression in these tested cell lines is correspondingly shown in Additional File 1: Figure S2A. To further evaluate the role of DNA methylation, IMR-32 cells were treated with 5-aza-2'-deoxycytidine (5-Aza), which appeared to suppress DNA methylation in *GSTp1 *and slightly decrease *Trip10 *DNA methylation in the first exon region (Figure 1C upper panel). In a good support of the MSP results, *Trip10 *mRNA levels were increased by 5-Aza in IMR-32 cells as compared to controls (Figure 1C lower panel), demonstrating that the *Trip10 *expression is regulated epigenetically and differentially by both DNA methylation and histone modification in a cell type-specific manner.

**Figure 2:**
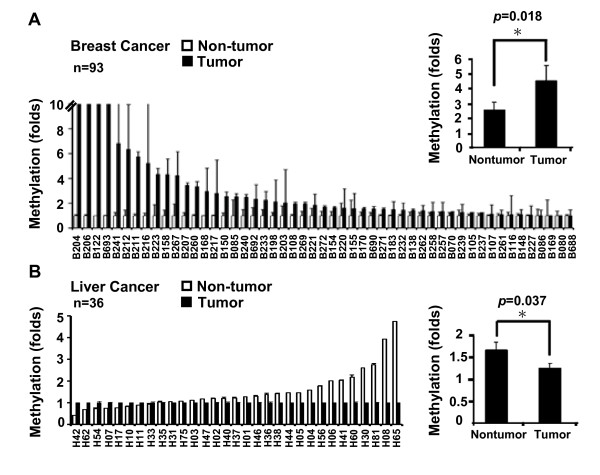
**Differential methylation of *Trip10 *in breast and liver cancers**. Representative DNA methylation of (A) breast cancer tissue and (B) liver cancer compared with adjacent non-tumor tissues. Results are expressed as mean and standard deviation. Breast cancer, n = 93 pairs; liver cancer, n = 36 pairs. *Analyzed by paired Student *t*-test.

To determine *Trip10 *methylation *in vivo*, we examined *Trip10 *promoter methylation in human breast cancer and liver cancer specimens and adjacent non-tumor tissues. As illustrated in Figure 2*Trip10 *was hypermethylated in breast cancer (Figure 2A), but hypomethylated in liver cancer (Figure 2B). Together, these data demonstrate that *Trip10 *is subject to epigenetic modification by DNA methylation in breast cancer and liver cancer tumorigenesis. Aberrant DNA methylation of *Trip10 *occurs *in vivo *and may contribute to neoplasm development.

### Trip10 interacts with Cdc42 and huntingtin in both IMR-32 and CP70 cells

**Figure 3:**
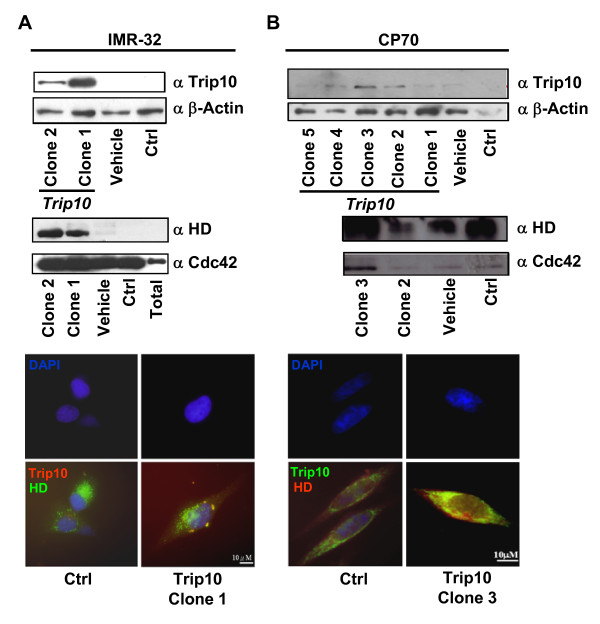
**Trip10 interacts with both Cdc42 and huntingtin (HD) and shows cell type-specific localization**. *Trip10 *was cloned and transfected into (A) IMR-32 cells and (B) CP70 cells; individual colonies were selected and analyzed by Western blot (top panels). Interactions of Trip10 with Cdc42 and HD were analyzed by immunoprecipitation. After immunoprecipitation of Trip10, the protein complex was probed with Cdc42 and HD antibodies (middle panels). Immunostaining (bottom panels) show the distribution of Trip10 and HD. Vehicle: empty vector only; Ctrl: transfection agent only.

Because *Trip10 *is differentially methylated in different types of cancer (Figure 1), we speculated that Trip10 functions in cell type-specific manner. *Trip10 *was thus cloned and overexpressed in IMR-32 and CP70 cells. Consistent with the qMSP results, endogenous Trip10 protein was undetectable in control IMR-32 cells by Western blot (Figure 3A, top), but weakly expressed in control CP70 cells (Figure 3B, top). Immunoprecipitation experiments showed that Cdc42, but not huntingtin, was expressed in IMR-32 cells (Figure 3A, center). In contrast, huntingtin was highly expressed in CP70 cells, whereas Cdc42 was expressed at low levels (Figure 3B, center). Overexpression of the *Trip10 *gene substantially increased cytosolic Trip10 protein and mRNA levels in both cell types (Figure 3 bottom, Additional File 1: Figure S2B). Moreover, huntingtin and Cdc42 were increased as well. Immunostaining results support the immunoprecipitation findings (Figure 3 bottom). These results demonstrate that Trip10 associates with Cdc42 and huntingtin in IMR-32 cells and CP70 cells, but the differential expression of these proteins may lead to activation of different signalling pathways.

### Trip10 promotes or suppresses in vitro colony formation and in vivo tumorigenesis in a cell type-dependent manner

**Figure 4:**
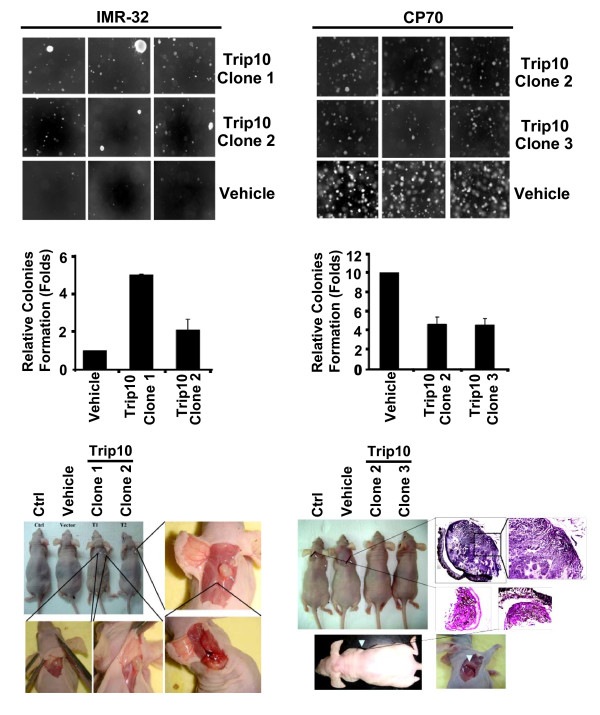
**Functional studies of Trip10**. (A) *Trip10 *overexpression in IMR-32 cells increased colony formation (top and middle left panels) and tumor growth in nude mice (bottom left). (B) In contrast, *Trip10 *overexpression in CP70 cells suppressed colony formation (right top and middle panels) and tumor growth in nude mice (each group, n = 6). Vehicle: empty vector only; Ctrl: transfection agent only.

Because Trip10 has been reported to regulate diverse functions and is differentially expressed in IMR-32 and CP70 cells, we next investigated the effects of overexpressed *Trip10 *in cell proliferation and survival. The soft agar assay was performed to evaluate *in vitro *colony formation. Overexpression of *Trip10 *promoted colony formation in IMR-32 cells (Figure 4A), but strongly inhibited colony formation in CP70 cells (Figure 4B). Both control and *Trip10*-overexpressing cells were then inoculated into nude mice to determine the *in vivo *effect of *Trip10 *on tumorigenesis. Consistent with results from the colony formation assay, IMR-32 cells overexpressing *Trip10 *formed tumors, some of which metastasized. In contrast, mice inoculated with control CP70 cells rapidly developed tumors, but tumors were not detected in mice inoculated with *Trip10*-overexpressing CP70 cells. These data demonstrate that Trip10 can either promote or inhibit tumorigenesis depending on the cell type in which it resides.

In Figure 3 we have demonstrated that Trip10 differentially associates with Cdc42 and huntingtin in IMR-32 cells and CP70 cells, we speculated that the differential expression of these proteins may lead to activation of different signalling pathways and contribute to the opposite oncogenic and tumor suppressive effect of Trip10. Because PI3K/Akt and MAPK pathways are often aberrantly activated in tumor cells, and they are reported to be associated with Cdc42 and huntingtin [12,23-25], thus we performed qRT-PCR to determine the mRNA expression of *Akt *and *MAPK14 *(encoding p38 MAPK) in *Trip10*-overexpressed CP70 and IMR-32 cells. Expression of *Akt*1, *Akt2*, and *MAPK14 *were elevated in *Trip10*-overexpressed cells, and the expression levels of these signalling components exhibited a positive correlation with endogenous *Trip10 *expression, in which more endogenous *Trip10 *expression is associated with greater *Akt1*, *Akt2*, and *MAPK14 *expression in CP70 cells as compared to the IMR-32 cells (Additional File 1: Figure S2B). Interestingly, *Akt3 *expression is much lower in CP70 than in IMR-32 cells, furthermore, overexpression of *Trip10 *increased *Akt3 *expression in IMR-32 cells, but not in CP70 cells. These data imply that distinct signalling components may have profound effect in the cell type-specific functions of Trip10.

## Discussion

Trip10 was initially identified as a Cdc42-interacting protein involved in GLUT4-mediated glucose uptake in adipocytes and muscle cells, but Trip10 is now known to have diverse functions in wide variety of cell types. We previously identified *Trip10 *as an ERα target gene [21]. In ER^+ ^breast tumor cells, DNA methylation of *Trip10 *was not detectable; however, disrupting ER signalling caused a time-dependent increase in DNA methylation of *Trip10 *and reduced mRNA levels [18]. *Trip10 *is consistently unmethylated in ER^+ ^breast tumors but hypermethylated in ER^- ^breast tumors. Because ER^- ^breast cancer is generally more malignant than ER^+ ^breast cancer, these data suggest that *Trip10 *hypermethylation promotes tumorigenesis. In the present study, we report that *Trip10 *expression is epigenetically regulated by DNA methylation and histone modification in a cell type-specific manner. Among the cell lines we examined, the DNA methylation level of *Trip10 *(from highest to lowest) was: brain tumor cells (IMR-32 and U87) > breast tumor cells (MCF7 and MDA-MB-231) > liver cancer cells (HepG2) > ovarian cancer cells (CP70) > MSCs (Figure 1A). Similar methylation patterns were observed in tumor specimens, *Trip10 *was hypermethylated in breast cancer but hypomethylated in liver cancer compared to adjacent non-tumor tissues (Figure 2). Interestingly, while the *Trip10 *promoter was methylated in IMR-32, MDA-MB-231, and HepG2 cells, several putative transcription factor binding sites (ER, AML-α, and CREB) were enriched for H3K4me3, association with H3K27me3 was contrarily low (Figure 1B). The expression levels of endogenous *Trip10 *mRNA in these cell lines (Additional File 1: Figure S2A) suggest that DNA methylation may interfere with H3K4me3 binding to the *Trip10 *promoter in these cells.

Functional assays reveal that Trip10 plays opposing roles in IMR-32 and CP70 cells, which may be due to differential expression of its interaction partners, thus activating different signalling pathways. The cellular localization of Trip10 also varies depending on the cell type. In COS7 and human macrophages, Trip10 is widely distributed in the cell in a "meshwork-like structure" [6]. In a skeletal muscle cell line, endogenous Trip10 is found in both the cytosol and perinuclear space, and its expression level is similar in immature myoblasts and differentiated myotubes [3]. In human brains, immunoexpression of Trip10 is detected in the nucleus and cytoplasm of neurons, activity and nuclear distribution are higher with more severe Huntington's disease [9].

In the present study, Trip10 was only sporadically in the cytosol and perinuclear region of IMR-32 control cells, but was more evenly distributed in the cytosol of CP70 control cells (Figure 3 immunostaining). Overexpression of *Trip10 *in IMR-32 cells caused Trip10 and huntingtin to colocalize and form perinuclear foci. In contrast, while overexpression of Trip10 in CP70 cells also increased huntingtin levels, both proteins remained in the cytosol without apparent foci formation. Western blot and immunoprecipitation studies revealed that both IMR-32 and CP70 cells express huntingtin and Cdc42, but Cdc42 was more strongly expressed in IMR-32 cells (Figure 3A), whereas huntingtin was more strongly expressed in CP70 cells (Figure 3B), even when *Trip10 *was overexpressed. Cdc42 is involved in migration; therefore, strong Cdc42 expression in IMR-32 cells may cause them to become more invasive, possibly explaining the enhanced *in vitro *colony formation and *in vivo *tumorigenesis and metastasis in mice inoculated with *Trip10*-overexpressing IMR-32 cells (Figure 4A). On the other hand, huntingtin increases cell death by promoting apoptosis. Thus, high levels of huntingtin in *Trip10*-overexpressing CP70 cells may lead to cell death, as shown by the lower rates of colony formation and tumorigenesis (Figure 4B).

Dysregulated signalling pathway is a key factor contributing to tumorigenesis and progression. In the present study, we found expression of endogenous *Akt*1, *Akt2 *and *p38 *correlates with endogenous *Trip10 *expression, in which greater *Trip10 *expression in CP70 cells is accompanied with more *Akt1/2 *and *p38 *expression in this cell type. Overexpression of *Trip10 *leads to concomitantly up-regulation of *Akt1/2 *and *p38 *in both cell types, implicating that both PI3K/Akt and p38 MAPK pathways are involved in Trip10-mediated cellular behaviours. Interestingly, *Akt3 *exhibits a distinct expression pattern. Expression of *Akt3 *mRNA is higher in IMR-32 cells as compared to CP70 cells. Overexpression of *Trip10 *only promotes *Akt3 *expression in IMR-32 cells but not in CP70, implicating that Akt3 may not be a key signalling component in CP70 cells, but may be important for tumorigenesis of IMR-32 cells. On the other hand, because amplification of *Akt3 *has also been reported in glioblastoma [26], we reason that elevated Akt3 expression may be crucial for brain tumor formation and progression. Functional studies of the three Akt family members have revealed that they are not redundant and each fulfills unique roles [27]. Thus lack of *Akt3 *expression along with high level of endogenous huntingtin in CP70 cells may be the determinant factors of Trip10-induced tumor suppression. In contrast, amplified Akt3 and Cdc42 may collaborate with Trip10 to trigger tumorigenesis In IMR-32 cells.

We do not rule out the possibility that specific isoforms of Trip10 are active in different cell types. In adipocytes, inactive Trip10 (CIP4/2) decreases Glut4 translocation to the plasma membrane [2], whereas in skeletal muscle cells, depletion of Trip10 (CIP4a) enhances insulin-stimulated glucose uptake by suppressing Glut4 endocytosis [3]. This difference can be explained, in part, by the fact that CIP4a does not contain the TC10-binding domain. Therefore, the differential effects of Trip10 in IMR-32 cells and CP70 cells may result from different isoforms in these two cell types, which recruit different interacting proteins. On the other hand, Trip10 directly interacts with WASP family verprolin-homologous protein (WAVE1) in a pancreatic cancer cell line and enhances its phosphorylation by the cytosolic tyrosine kinase c-Abl [11]. Trip10 itself is also subject to phosphorylation by c-Abl and dephosphorylation protein tyrosine phosphatase containing a PEST domain (PTP-PEST) [11]. Thus IMR-32 and CP70 cells may be equipped with different signaling pathways to regulate Trip10 activity and function.

Taken together, our data demonstrate that *Trip10 *expression is regulated by both DNA methylation and H3K4me3. Trip10 can enhance tumorigenesis or act as tumor suppressor depending on the cell type in which it is expressed.

## Conclusions

Here we report that *Trip10 *is differentially methylated in different types of cancer cell lines and tumors. Analysis of histone modification in MDA-MB-231, HepG2, and IMR-32 cells demonstrated that *Trip10 *is associated with H3K4me3, but not H3K27me3. Trip10 can be oncogenic or tumor suppressive, increasing IMR-32 cell proliferation and inhibiting CP70 cell proliferation. The cell type-specific effect may be due, in part, to different cellular signalling partners recruited by Trip10.

## Abbreviations

Trip10: thyroid hormone receptor interactor 10; MSC: mesenchymal stem cell; 5-Aza: 5-aza-2'-deoxycytidine; H3K27me3: histone 3 lysine 27 trimethylation; H3K4me3: histone 3 lysine 4 trimethylation.

## Competing interests

The authors declare that they have no competing interests.

## Authors' contributions

YWL and SHH designed the study and drafted the manuscript. CCH, YWL, YLL and YCH carried out the MSP and bisulfite sequencing. CCH carried out the ChIP PCR. MJT cloned the human *Trip10*. TYK and JYY participated in immunoprecipitation and immunostaining. CCH and WSS carried out colony formation assay. CMC, PYC and KTU performed the immunohistochemistry. PSY, YSC, and THH helped to draft the manuscript. All authors read and approved the final manuscript.

## Supplementary Material

Additional file 1 **Supplementary materials**. Additional file contains the supplementary materials which include: Supplementary Figures S1 to S2 and Supplementary Table S1. Click here for file
