# Supplementary material for: Functional characterization of Trip10 in cancer cell growth and survival
Source: J Biomed Sci. 2011 Feb 7;18(1):12. doi: 10.1186/1423-0127-18-12 (PMC3044094; doi:10.1186/1423-0127-18-12)
Supplement: Additional file 1 — Supplementary materials. Additional file contains the supplementary materials which include: Supplementary Figures S1 to S2 and Supplementary Table S1. [file 1423-0127-18-12-S1.PDF]

Additional File

Functional characterization of *Trip10* in cancer cell growth and survival

Chia-Chen Hsu, Yu-Wei Leu, Min-Jen Tseng, Kuan-Der Lee, Tzen-Yu Kuo, Jia-Yi Yen, Yen-Ling Lai, Yi-Chen Hung, Wei-Sheng Sun, Chien-Min Chen, Pei-Yi Chu, Kun-Tu Yeh, Pearlly S. Yan, Yu-Sun Chang, Tim H.-M. Huang, and Shu-Huei Hsiao

This file includes:

Supplementary Figures S1 to S2
Supplementary Table S1

Supplementary Figure Legend

Supplementary Figure S1 Bisulfite sequencing of the *Trip10* promoter. This figure displays the same data shown in Figure 1A, but with detailed information about the eight clones of each cell line. The CpG locations are shown as vertical bars in the promoter and first exon of *Trip10*. Arrows indicate the location of methylation-specific polymerase chain reaction (MSP) primers. Open circles indicate unmethylated CpG sites and circles filled to varying degrees show the extent of methylation at specific CpG sites in the cell, given as percentage on the right.

Supplementary Figure S2 Expression of *Trip10*, *Akt* isoforms and *p38* in diverse cell types. (A) Expression of endogenous *Trip10* mRNAs in cancer cell lines and MSCs. (B) Expression levels of *Trip10*, *Akt1-3* and *MAPK14* (*p38*) in *Trip10*-overexpressed cells. Ctrl: cells treated with transfection agent only; Vehicle: cells treated with empty vector only; Trip10: cells were transfected with cloned *Trip10*. Data were obtained by qRT-PCR.

Supplementary Figure S1

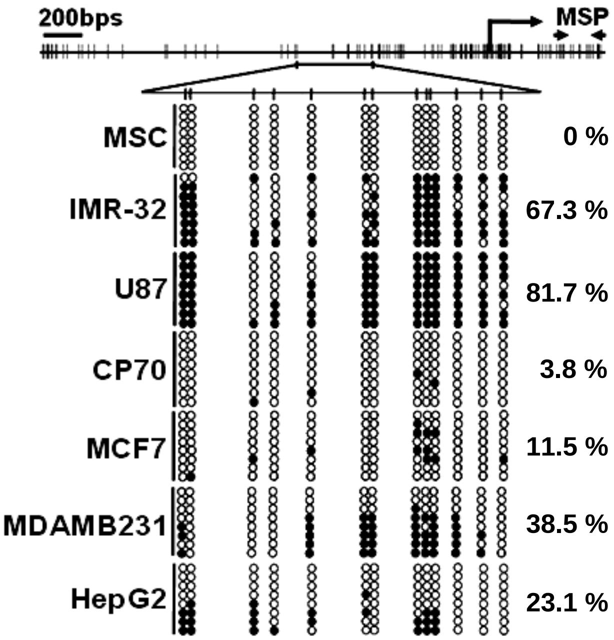

(A)

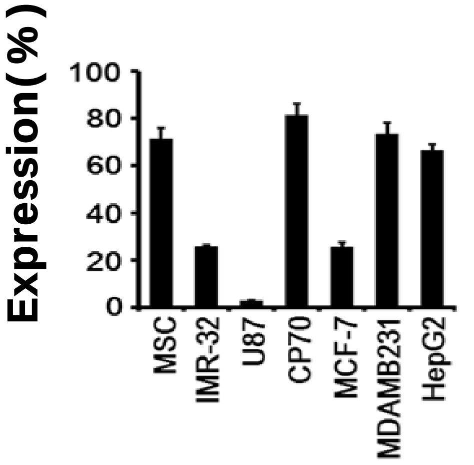
Expression(%)
100
80
60
40
20
0
MSC
IMR-32
U87
CP70
MCF-7
MDAMB231
HepG2

(B)

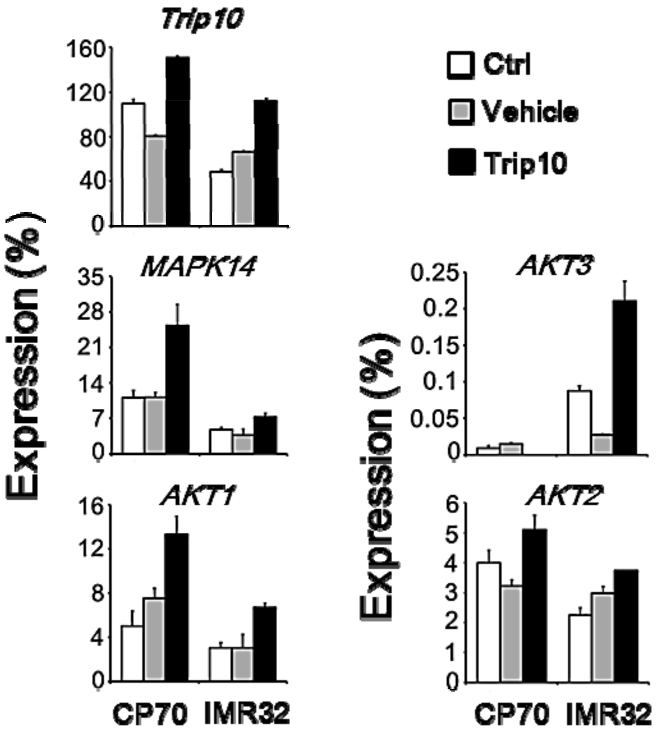
Trip10
Ctrl
Vehicle
Trip10
Expression (%)
MAPK14
AKT3
AKT1
AKT2
CP70
IMR32

Supplementary Table

Supplementary Table S1. Primer sequences.

Primer	Gene	Sequence(5'→3')	Detection
Trip10_MSP_F	Human *Trip10* (NM_004240)	GAAGTTTATTTGGGAGTTTTAGTACGT	MSP
Trip10_MS_R		AATAACCTCTCTCAACCGCC	
Trip10_bis_F		TTAGGTTAAATTGGAAAATGGGTAG	Bisulfite Sequencing
Trip10_bis_R		TCCCACTAATAATCTAATCCCTCC	
Trip10_RT_F		TGATGAGGATTTCGAGGAGGAACC	RT PCR
Trip10_RT_R		GGGTTCAATTGAGCGTGACTCGGA	
Trip10_Cloning_F		ATAAGCTTACGATTGGGGCACTGAGCTGTGG	Trip10 Cloning
Trip10_Cloning_R		CTAAGCTTTGGCAGGGTTCAATTGAGCGTG	
Trip10_ChIP_ER_F		GGTTGAGGAAGGACTTCTTGAGT	ChIP PCR
Trip10_ChIP_ER_R		CTTAAAGAACCCCCACCCATTT	
Trip10_ChIP_CREB_F		CTCACTCTCACCAATCGCTATAAA	ChIP PCR
Trip10_ChIP_CREB_R		GGGATACTGGGAAAGGTAGTTTATTT	
Trip10_ChIP_AML1a_F		AACTCTGACCCCAGGTGAACTC	ChIP PCR
Trip10_ChIP_AML1a_R		GGATAGAGGCCAGAGGCTTAAC	
GAPDH_RT_F	*GAPDH* (NM_002046)	CCCCTTCATTGACCTCAACTAGAT	RT PCR Control
GAPDH_RT_R		CGCTCCTGGAAGATGGTGA	
Col2A1_MSP_F	*Col2A1* (NM_0033150)	TCTAACAATTATAAACTCCAACCACCAA	MSP Control
Col2A1_MSP_R		GGGAAGATGGGATAGAAGGGAATAT	
bActin_F	*ACTB* (NM_001101)	TCACCCACACTGTGCCCATCTACGA	ChIP PCR Control
bActin_R		CAGCGGAACCGCTCATTGCCAATGG	
MAPK14_a_RT_F	Human *p38* (NM_131722)	TCGACTTGCTGGAGAAGATGCTTGT	RT PCR
MAPK14_a_RT_R		CAGGACTCCATCTCTTCTTGGTCAA	
Akt1_RT_F	Human *AKT1* (NM_005163)	CTCACAGCCCTGAAGTACTCTTTCCA	RT PCR
Akt1_RT_R		TCCAGCATGAGGTTCTCCAGCTTGA	
Akt2_RT_F	Human *AKT2* (NM_001626)	ATGGTCGCCAACAGCCTCAAGCA	RT PCR
Akt2_RT_R		GAGATAGTCGAAGTCATTCATGGTCAC	
Akt3_RT_F	Human *AKT3* (NM_005465)	GATTGTGTACCGTGATCTCAAGTTGGAGA	RT PCR
Akt3_RT_R		GCTCGGCCATAGTCATTATCTTCTAACA	
